# Driving Hydrolysis and Acetolysis of Poly(ethylene terephthalate) (PET) by Microwave and Thermal Energy Inputs: A Comparative Study

**DOI:** 10.1021/acsomega.5c12098

**Published:** 2026-02-23

**Authors:** Patrícia Pereira, Ashley C. Daniszewski, Matthew Staack, Emir Salmanzadeh, Hilal Ezgi Toraman, Jianli Hu, Christian W. Pester, Phillip E. Savage

**Affiliations:** † Waltemeyer Department of Chemical Engineering, 8082 The Pennsylvania State University, University Park, Pennsylvania 16802, United States; ‡ Department of Chemical and Biomedical Engineering, 5631 West Virginia University, Morgantown, West Virginia 26505, United States; § Department of Energy and Mineral Engineering, The Pennsylvania State University, University Park, Pennsylvania 16802, United States; ∥ Institute of Energy and the Environment, The Pennsylvania State University, University Park, Pennsylvania 16801, United States; ⊥ Department of Materials Science and Engineering, 5972 University of Delaware, Newark, Delaware 19716, United States

## Abstract

This study compares microwave and conventional thermal energy inputs for the hydrolysis and acetolysis of both virgin and postconsumer poly­(ethylene terephthalate) (PET). Reaction conditions in these experiments range from 200 to 300 °C and from 5 to 90 min. In no instances did the yields of terephthalic acid monomer or incompletely depolymerized PET demonstrate statistically significant or practically significant differences with these two different energy inputs. For fixed reaction conditions, yields of terephthalic acid were comparable from both methods, regardless of whether the reaction medium was water, acetic acid, or a mixture of the two. The visual appearance of the unreacted plastic was likewise the same for microwave and conventional thermal energy inputs when using identical solvents. These results suggest that the bulk fluid temperature is the controlling factor for PET depolymerization in a homogeneous fluid phase of water and/or acetic acid with no added catalyst. The reacting system responds similarly whether the heating is via microwave irradiation or via conduction across the reactor wall.

## Graphical abstract

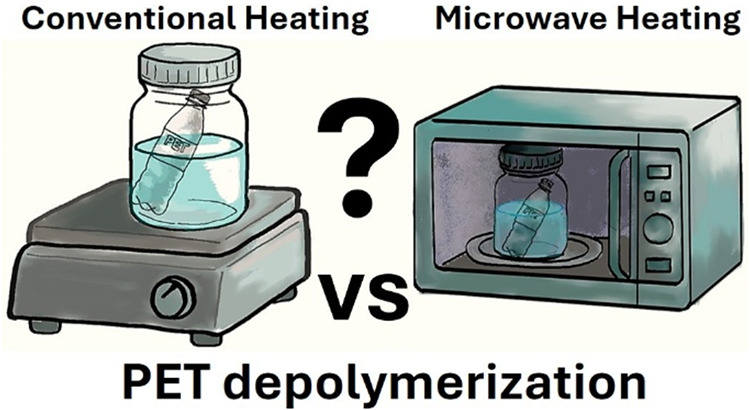

## 1 Introduction

Poly­(ethylene terephthalate) (PET) is widely used in disposable bottles and in fast fashion. Designed to be discarded, these products quickly find their way to waste streams. PET recycling remains a challenge globally, with only 29% of collected PET bottles being recycled.^1^ While mechanical recycling is the approach widely applied, solvolytic chemical recycling approaches, such as hydrolysis and acetolysis, however, offer a viable alternative. Water and acetic acid can serve as both the reaction medium and as a reactant. Both molecules react with the ester linkages that connect the repeat units of PET to produce the monomer terephthalic acid (TPA) along with ethylene glycol (for hydrolysis) or ethylene glycol diacetate (EGDA) (for acetolysis). In concept, the monomers could then be used to synthesize virgin PET anew, thereby facilitating a circular life cycle for this material.

PET hydrolysis has been widely studied^2^ but PET acetolysis only recently so.^3–6^ The large majority of prior studies with both solvents used chemical reactors wherein the reaction was driven by conduction of thermal energy through a solid reactor wall. Microwave energy, on the other hand, can heat the reactor fluid phase directly and eliminate the wall resistance that impedes conventional thermal energy transfer.^7^ This approach for energy delivery may provide opportunities for process intensification.

Microwave heating relies on mechanisms such as dipolar polarization and ionic conduction, via molecular friction and energy dissipation from ion movement under electric fields.^8^ The electricity required for microwave heating can, in principle, be sourced from renewable energy, emphasizing its potential for a lower carbon footprint as renewables contribute a larger portion of the electrical grid. PET has a low microwave absorption, but water and acetic acid efficiently convert microwave energy into thermal energy, which facilitates indirect heating of PET in a water-acetic acid solvent system.^9^

There have been a few prior studies on microwave-driven hydrolysis of PET^10–18^ without any added catalyst. However, to the best of our knowledge, studies on microwave-driven acetolysis remain unprecedented. The prior studies for PET hydrolysis driven by thermal energy and microwave energy were almost always done using just one of the methods alone. Of the prior work on uncatalyzed microwave-driven hydrolysis of PET, only Ikenaga et al.^11^ directly compared microwave-driven and thermally driven PET hydrolysis. The authors used microwave energy for both cases, with a glass reactor wall to study microwave heating and a silicon carbide (SiC) reactor wall to study conventional heating, respectively. Glass is transparent to microwaves, but SiC is a strong microwave absorber. It would be heated by the radiation and then transfer heat from the walls by conduction and convection to the reaction solution. For three sets of comparable reaction conditions, the microwave heating (glass reactor) gave higher TPA yields. For example, PET hydrolysis for 15 min at 231 °C (29 bar) gave 34.7% TPA yield with the glass reactors (microwave heating) and just 6.1% with the SiC reactors (conventional heating). The authors suggested the influence of microwaves on PET depolymerization rates could be due to enhanced diffusion of water into the PET matrix. They used a 3/1 w/w loading of water/PET, which is much more concentrated in PET when compared to other studies. Additionally, the TPA yield was quantified gravimetrically, rather than chromatographically or spectroscopically, which can overestimate the amount formed.^19^ Regrettably, the absence of experimental uncertainties for the product yields and detailed temperature–time data for the reactors make it impossible to determine the statistical significance of the differences in TPA yields reported.

A second report^13^ asserts that microwave-driven hydrolysis with no added catalyst caused the PET to decompose at one rate during the initial stages of the reaction and at a different, faster rate at longer times. The authors attributed this difference in apparent pseudo-first order kinetics during different temporal regions to nonthermal effects from the microwave radiation. An alternate explanation, though, is that the kinetics simply reflected the autocatalysis that occurs during PET hydrolysis. The rate would be slow initially and then accelerate as the concentration of TPA, a known PET hydrolysis catalyst, increases. None of the other reports provide direct comparisons or offer explanations for the role the microwaves may be having during the neutral hydrolysis reaction.

In contrast to uncatalyzed hydrolysis, there have been more previous studies on microwave-driven PET hydrolysis in the presence of acid, alkaline, and solid catalysts.^2^ These catalytic studies are outside the scope of the present work, which focuses on the simpler system of PET in water, acetic acid, or their mixture, with no added catalyst. There have also been prior direct comparisons of microwave-driven and conventional thermal-energy-driven solvolytic depolymerization of PET, especially for glycolysis (ethylene glycol as solvent and reactant) and again with catalysts. Pingale and Shulka^20^ found the yield of bis­(2-hydroxyethyl) terephthalate (BHET) monomer was the same for microwave and conventional heating (for glycolysis catalyzed by zinc and sodium salts), but the reaction time needed was reduced by an order of magnitude. Scé et al.^21^ reported similar results for glycolysis catalyzed by ionic liquids. Selvam et al.^22^ also found microwave-driven depolymerization to be faster (for glycolysis heterogeneously catalyzed by ZnO), but only because it provided more rapid heat up to the set point temperature than did the reactor with conventional heating. Another study^23^ showed microwave heating accelerated PET glycolysis catalyzed by titanate nanotubes. In these studies with heterogeneous catalysts, the catalyst itself can be a microwave absorber and thereby accelerate reaction rates via localized heating of the catalyst surface. These studies employed catalysts and ethylene glycol as solvent, so they do not address the present question of the influence of microwave energy on PET hydrolysis/acetolysis without added catalyst, relative to conventional thermal heating.

The present study aims to directly compare results from microwave-driven and conventional thermally driven depolymerization of PET in water, acetic acid or their mixture with no added catalyst. By examining these processes under equivalent reaction conditions and by applying standardized methods (e.g., severity index, yield vs time, Arrhenius plot) with both our experimental data and published data, we deliver the first broad comparison of yields and rates for microwave vs conventional heating for hydrolysis/acetolysis of PET with no added catalyst. This comparison constitutes the major component of the novelty of this work, but additional novelty accompanies the new reaction conditions and data we report herein. More specifically, this study presents new data for PET hydrolysis and acetolysis with both conventional heating and microwave irradiation at the same reaction conditions. It also presents additional new data for PET hydrolysis with microwave irradiation and for PET hydrolysis with conventional heating. Reaction conditions range from 200 to 300 °C and from 5 to 90 min.

## 2 Materials and Methods

### 2.1 Materials

Perrier sparkling water bottles (16.9 oz) served as the source of postconsumer PET after the removal of labels and caps, cleaning, and cutting into small chips (6 ± 2 mm × 8 ± 2 mm, with a thickness 0.5 mm for the body and 2 mm for the base of the bottle). We reported more on the characterization of the chips previously.^24^ This material was used in conventional heating experiments and in microwave experiments at West Virginia University. Small cylindrical pellets of virgin PET (Shaw, Inc.) with mean mass of 17.1 ± 1.2 mg were used as received in microwave experiments at the Pennsylvania State University. The length and diameter of the pellets were nearly equal at about 2.5 mm. Differential scanning calorimetry gave the melting temperature as 243.7 °C. TPA was obtained from *Sigma-Aldrich*, dimethyl sulfoxide was from *Millipore Sigma*, and glacial acetic acid (AcOH) was from *Fisher Scientific*. Deionized water was obtained through an in-house water purification system.

### 2.2 PET Solvolysis Experimental Method

PET hydrolysis with conventional heating was performed in a stainless-steel *Swagelok* reactor (1/2-in. nominal size) with 4 mL volume. The reactor loading was such that 95% of the volume would be occupied by the fluid phase at reaction conditions. For example, for hydrolysis at 200 °C, this meant 3.29 mL of water was loaded into the reactor. The liquid/PET w/w loading was 10/1. Experiments with water-acetic acid mixtures at 200 °C used 2.2 mL water loading and 25%, 50% or 80% acetic acid in relation to the water volume (20, 33, and 44 vol % AcOH, respectively). Acetolysis at 200 °C used a 3.29 mL acetic acid loading and no water. The reactors were heated for 1–2 h in an isothermal Techne sand bath operating at the desired hydrolysis temperature (200–245 °C) and then quenched in room-temperature water to terminate the reaction.

Microwave-driven hydrolysis of PET at West Virginia University (WVU) was performed in a 100 mL Teflon *CEM EasyPrep* vessel equipped with a fiber optic probe and IR sensor for continuous monitoring and temperature control. The reactor was loaded with water (41.09 mL for a reaction at 200 °C), a 10/1 liquid/PET w/w ratio, and then sealed. Experiments with acetic acid at 200 °C used a 27.5 mL water loading and 25%, 50%, or 80% acetic acid relative to the water volume (20, 33, and 44 vol % AcOH, respectively). Acetolysis at 200 °C used a 41.09 mL acetic acid loading. The vessel was placed in a *CEM MARS 6 Synthesis* microwave reactor (2.45 GHz, up to 1800 W) on a rotating carousel. The reactor was heated to the desired set point (200–245 °C) over 20 min and then held at the set point for 1–2 h. Figure 1 shows temperature, pressure, and power profiles during the reaction. Cooling required at least 15 min.

**Figure 1:**
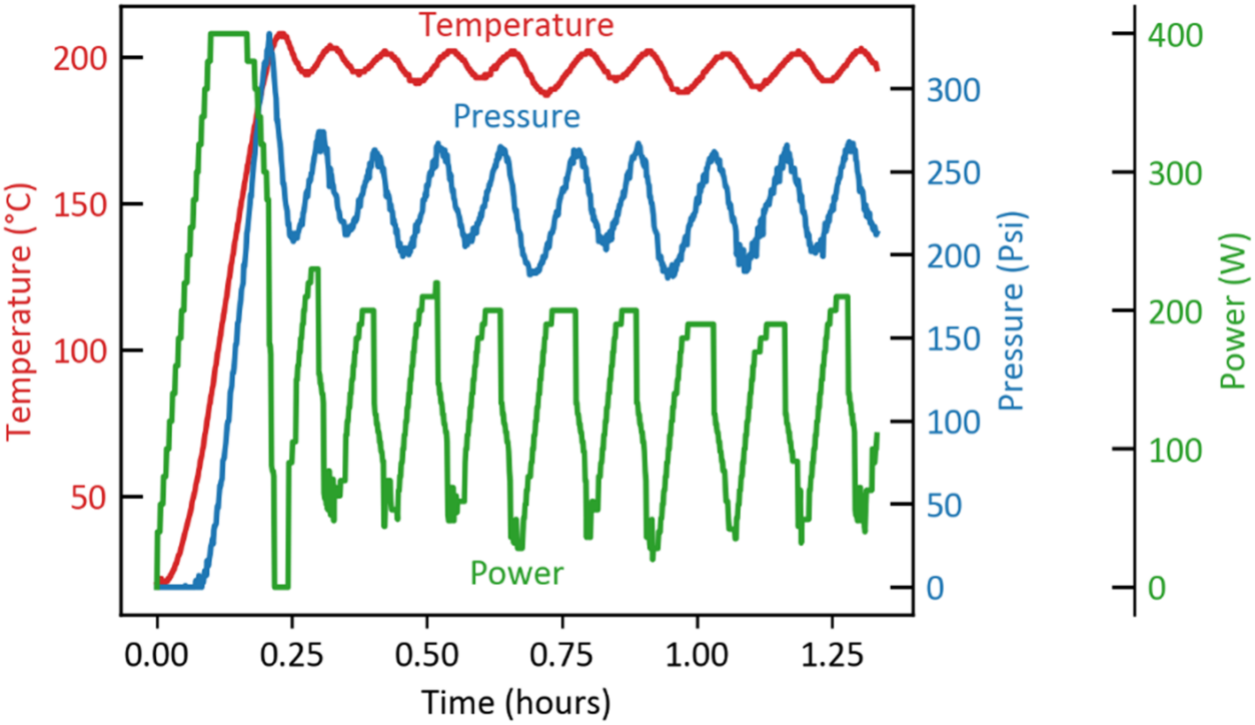
Typical variation of temperature, pressure, and power in WVU microwave reactor for a 200 °C set point temperature.

This microwave-driven hydrolysis setup at WVU requires a 20 min heating ramp, whereas it is just 3 min in the mini-batch reactors used in experiments with conventional heating. To determine how much depolymerization occurred during this 20 min heat-up time, we conducted control experiments in the microwave system at a set point temperature of 200 °C. Microwave reactors were loaded with PET and water, placed in the microwave system, and then removed immediately after the 20 min heat-up period. These experiments gave no detectable TPA yield, but we cannot rule out morphological or crystallinity changes in the PET possibly differing during the different heat-up regimens.

Microwave-driven hydrolysis of PET was performed at the Pennsylvania State University in an 80 mL quartz reaction vessel. The reactors were loaded with 1.2 g of virgin PET pellets, 12 mL of deionized water, and a stir bar for mixing (∼300–500 rpm). The reactors were placed into the microwave reactor (Multiwave 5000, Anton Paar), operating at 2.45 GHz with a maximum power of 1800 W. Maximum power was used to reach the set point temperature as quickly as possible. Afterward, the power was controlled to maintain the set point temperature for the nominally isothermal reaction for the specified duration. Finally, the products were cooled to 65 °C inside the microwave reactor. Control experiments showed no TPA formation during the heating ramp for set-point temperatures below 300 °C. The run at 300 °C showed 29% PET conversion and 3% TPA yield during the heating to the set-point temperature.

Regardless of the system used, three independent runs were performed for each experimental condition. Mean values were taken as the best estimates for product yields. Standard deviations were calculated to estimate the run-to-run variability in each product yield.

### 2.3 Product Recovery and Analysis

To recover the reactor contents, deionized water was added to the reactors, mixed with the reaction products, and withdrawn. The aqueous and solid phases were separated by either filtration or centrifugation. When using filtration, the filters and related accessories and the reactors were dried at 80 °C overnight, and the solid material was recovered as dried solids (composed of residual PET and oligomers formed, TPA, and byproducts). When using centrifugation, the wet solids were dried in an oven at 45 °C.

DMSO was added to the dried solids to dissolve any TPA present. The resulting solution was then passed through PTFE membrane filters, with a diameter of 25 mm and a pore size of 1 μm, attached to a syringe. The undissolved solids (US) remaining on the filter consisted of any unconverted PET along with DMSO-insoluble oligomers and byproducts. These solids were dried and weighed. The yield (wt % based on PET mass loaded into the reactor) of these undissolved solids was taken to be the yield of unconverted PET.

The DMSO solution was analyzed via a *Shimadzu* High-Performance Liquid Chromatograph (HPLC), with a *Waters* reverse-phase symmetry C18 column (5 μm particle size, 150 mm × 4.6 mm) at 40 °C, and an *SPD-M20A* photodiode detector at 240 nm. The mobile phase consisted of HPLC-grade acetonitrile at 0.1 mL/min, combined with a mixture of 0.1 vol % formic acid aqueous solution at 0.3 mL/min. Each sample analysis had an injection volume of 1 μL. The same reaction products were observed from both conventional and microwave heating. Along with TPA, identified products included smaller amounts of bis­(2-hydroxyethyl) terephthalate (BHET) and mono (2-hydroxyethyl) terephthalate (MHET). Analysis of standard solutions containing known concentrations of TPA in DMSO provided a calibration curve. The TPA yield was determined by the ratio of the mass of TPA produced (*m*_TPA_) to the maximum TPA mass available for production from the initial PET loading as

1 $$Y_{\mathrm{TPA}}\;\left(\%\right)=\frac{m_{\mathrm{TPA}}}{m_{\mathrm{PET}}\times 0.86}\times 100$$

where 0.86 reflects the stoichiometry of the hydrolysis reaction. Complete hydrolysis of a given mass of pure PET (*m*_PET_) would give 86% of that mass in TPA and the balance would be ethylene glycol.

## 3 Results and Discussion

### 3.1 PET hydrolysis—Comparing Yields from Identical Experimental Conditions

PET hydrolysis experiments with conventional heating were conducted at the Pennsylvania State University and experiments at identical, carefully controlled reaction conditions were conducted at WVU under microwave irradiation. The experiments used PET chips from a postconsumer bottle and were conducted at isothermal temperatures of 200 to 245 °C for 60 min at saturation pressures. Any differences in product yields would be due solely to the heating modality.

Figure 2 shows the yields of TPA and unconverted PET from PET hydrolysis with microwave irradiation and conventional heating at identical reaction conditions. The yields of TPA increased with temperature, as expected, and were very similar at each temperature investigated, regardless of how energy was provided. Likewise, the yields of PET at a given temperature were very similar, whether conventional heating or microwave irradiation was used.

**Figure 2:**
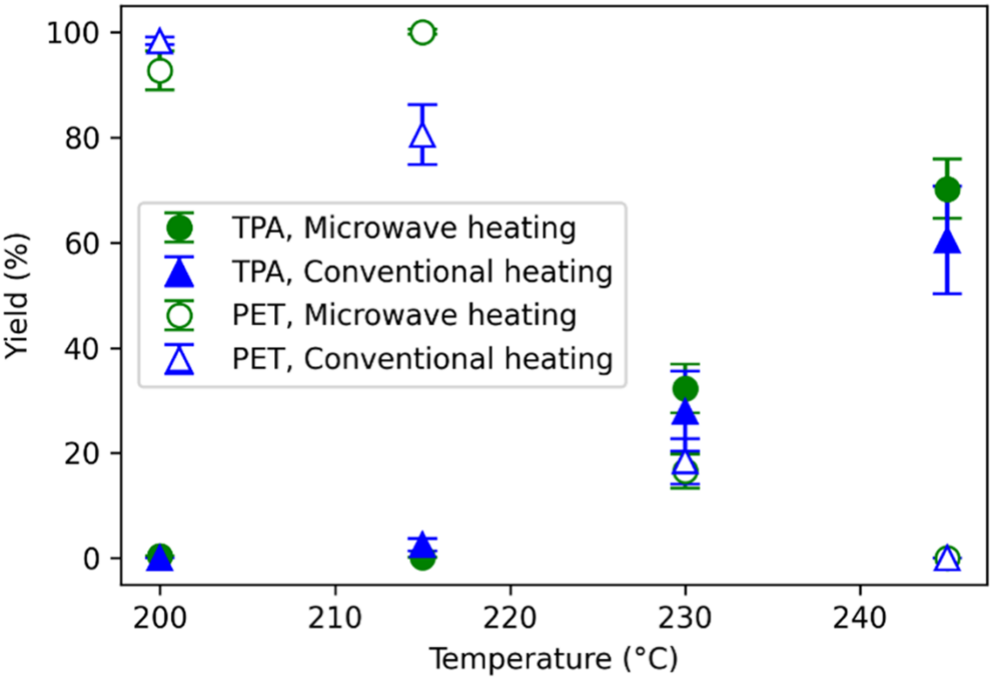
Yields of TPA and PET from PET neutral hydrolysis with no added catalyst for 60 min with conventional heating or microwave irradiation and water/PET w/w loading of 10/1.

To determine more rigorously whether the product yields under microwave and conventional heating were meaningfully different, we applied the Welch two one-sided equivalence test (TOST) with an arbitrary ±10 percentage point (pp) margin (α = 0.05; *n* = 3 per condition), alongside a standard two-side *t* test. Equivalence of the TPA yields produced with the two methods was confirmed for hydrolysis at 200 °C (90% CI 0.23–0.57, pTOST = 3.6 × 10^–5^) and at 215 °C (90% CI −4.5 to −1.1, pTOST = 6.4 × 10^–3^). For hydrolysis at 230 °C (90% CI −8.1 to +16.5) and 245 °C (90% CI −7.2 to +27.6), the mean TPA yields were not statistically different but the wider confidence intervals (CI) prevented a statistical determination of equivalence. The yields of PET from hydrolysis at 200 °C were not statistically different, but equivalence could not be concluded because the CI slightly exceeded the −10 pp margin. The yields from hydrolysis at 215 °C presented no equivalence. The yields from hydrolysis at 230 °C (90% CI −7.9 to +5.9) and at 245 °C were statistically equivalent. Thus, across the tested temperatures, the product yields from microwave and conventional heating were nearly always statistically equivalent. It appears the microwave radiation is simply heating the reaction medium, and that whether the heating is via conduction of thermal energy through a reactor wall or microwave heating exerts no statistically significant influence on product yields from the neutral hydrolysis of PET at the tested conditions. Note the reaction conditions used in Figure 2 provided opportunities to observe this system at low, moderate, and essentially complete PET conversion.

### 3.2 PET Hydrolysis—Comparing Results from Different Experimental Conditions

We wish to use the few prior reports on uncatalyzed PET hydrolysis driven by microwave irradiation for additional comparisons with results from conventional heating. Two such articles were discussed in the introduction section, and the remaining microwave studies on PET hydrolysis with no added catalyst are summarized here.

Liu et al.^12^ used a 10/1 w/w ratio of water/PET in their study of microwave hydrolysis. They controlled the reactor pressure (not temperature). The temperature can be inferred from thermodynamics as the saturation temperature at the given pressure. They report complete depolymerization of PET at 20 bar (*T*_sat_ = 212 °C) and 120 min. This account did not provide the reactor heat-up time, and it quantified the TPA yield gravimetrically, an approach that can overestimate the true yield^24^ due to the presence of similar byproducts. We use the data for PET conversion (but not TPA yield) from this study in our broader comparison. Wang et al.^16^ also provide data for PET conversion (at 175, 180, and 185 °C) from microwave-driven hydrolysis with a 10/1 w/w loading of water/PET.

Follow up work from this same lab^15,17^ investigated catalyzed hydrolysis of PET driven by microwave irradiation. Yields of products were again determined gravimetrically. A control experiment with uncatalyzed hydrolysis led to a PET conversion of 65.5% from reaction at 220 °C, 210 min, with a 10/1 w/w ratio of water/PET. Another control experiment provided 59.3% conversion at 200 °C, 210 min and a 10/1 w/w water/PET ratio. There was no comparison of outcomes from microwave-driven vs conventionally heated reactions. We use the data for PET conversion from these studies in our broader comparison.

Allaf et al.^14^ used microwave irradiation to hydrolyze PET, as part of a study into the effects of various PET pretreatments. The average yield of TPA at nominal conditions of 220 °C for 30 min was 60%. The TPA yield was determined gravimetrically (not chromatographically), however, so we exclude these few data points from the present comparisons. This article provides no comparison of outcomes from conventional heating vs microwave irradiation.

Kang et al.^10^ examined PET hydrolysis catalyzed by zeolites, but they provide some data from control experiments with uncatalyzed hydrolysis. These authors used a very high water/PET w/w reactor loading ratio of 120 and showed this loading ratio had a large influence on their results. They reported that the PET conversion for catalyzed hydrolysis was much lower (22% vs 99%) at water/PET w/w loading ratios around 10, as used more typically and in the present study. They reported the activation energy for uncatalyzed hydrolysis of PET to be 19.5 kJ/mol, a value which is far lower than those reported in other studies, where the activation energy is around 100 kJ/mol.^24^ There also seems to be an inconsistency between the rate constant values they report on their Figure 4b and the values used on the Arrhenius plot in Figure 4c. Finally, this article reports data in the Supporting Information showing TPA yields greatly exceeding the corresponding PET conversions, which is physically impossible. Given these issues, we replicated their control experiment with no catalyst. We performed neutral hydrolysis of PET in the Penn State microwave reactor, which was the same model used in their study, at the same stated conditions (rapid heating to the set point temperature of 230 °C, 30 min holding time) and with the same 120/1 water/PET w/w loading (0.1 g PET and 12 mL water). We found a TPA yield of just 0.04 ± 0.03%, whereas Kang et al. reported a much higher yield of 77%. The PET yield in our experiment was 96.2 ± 6.6%, indicating the conversion was not statistically different from zero. Given the issues with the data in this article, the much higher water/PET loading used, and the divergence of their results from ours, we have excluded these data from this broader comparison.

We have also conducted additional PET hydrolysis experiments with both conventional heating and microwave irradiation (in addition to those in Figure 2) at Penn State. This work in a microwave reactor system employed temperatures higher than those examined in prior published microwave studies or in the microwave reactor data in Figure 2. At these higher temperatures (*T* > 250 °C) PET would be above its melting point and exist in a molten state in the reactor. Table 1 summarizes this additional new data from the present work.

**Table 1:** Yields of TPA and Unconverted PET from Hydrolysis of PET with no Added Catalyst and a water/PET w/w loading of 10/1

nominal reaction conditions	energy input	TPA yield (%)	PET yield (%)	Pseudo-1st order ln(*k* [s^–1^])
200 °C, 30 min	microwave	1.9 ± 0.4	82.3 ± 7.3	–9.1
230 °C, 30 min	microwave	0.1 ± 0.2	98.0 ± 2.9	–11.4
245 °C, 30 min	microwave	8.5 ± 3.2	50.0 ± 7.2	–7.9
260 °C, 30 min	microwave	28.9 ± 5.7	8.8 ± 4.1	–6.6
270 °C, 15 min	microwave	14.8 ± 4.0	25.7 ± 5.5	–6.5
300 °C, 0 min	microwave	3.0 ± 0.6	71.0 ± 2.2	–
300 °C, 5 min	microwave	28.0 ± 4.0	3.4 ± 1.5	–4.6
300 °C, 15 min	microwave	79.6 ± 2.9	0.9 ± 0.1	–5.3
300 °C, 30 min	microwave	92.4 ± 1.3	1.0 ± 0.1	–6.0
240 °C, 15 min	thermal	0 ± 0	85.8 ± 1.6	–8.7
240 °C, 60 min	thermal	77 ± 2		
230 °C, 20 min	thermal	0 ± 0		
230 °C, 40 min	thermal	8 ± 3		
220 °C, 60 min	thermal	38 ± 7		
220 °C, 90 min	thermal	61 ± 6		

Table S1 in the Supporting Information provides literature data for TPA and/or PET yields from PET hydrolysis with no added catalyst that we also use for comparison. To ensure all comparisons of yields will be made on a consistent basis, we confine the data in Table S1 to studies that determined the fraction of unconverted PET gravimetrically, the yield of TPA spectroscopically or chromatographically, and used water/PET w/w loadings between 8/1 and 10/1, as this ratio can have a strong influence on product yields.^10,24^

#### 3.2.1 PET Hydrolysis—Comparing Rate Constants for Isothermal Depolymerization

Monitoring the PET yield at different reaction times permits calculation of pseudo-first-order rate constants for PET conversion. This kinetics approach provides a way to compare results from the different studies of PET hydrolysis via conventional and microwave heating. We recognize the hydrolysis kinetics are not truly first order, as the reaction is autocatalytic. We use the first-order treatment as a simple and useful approximation.

Figure 3 displays the Arrhenius plot, constructed from the data in Figure 2, Tables 1, and S1 for batch holding times long enough to consider the reaction to be isothermal. There is variability for the pseudo-first order rate constants calculated at any given temperature, as would be expected for analysis of an autocatalytic system and with any compilation of data from many different studies and different laboratories. The data are consistent, however, in showing the rate constants arising from hydrolysis with microwave heating, both from this present work and literature, being interspersed within the same region as the rate constants from PET hydrolysis via conventional heating.

**Figure 3:**
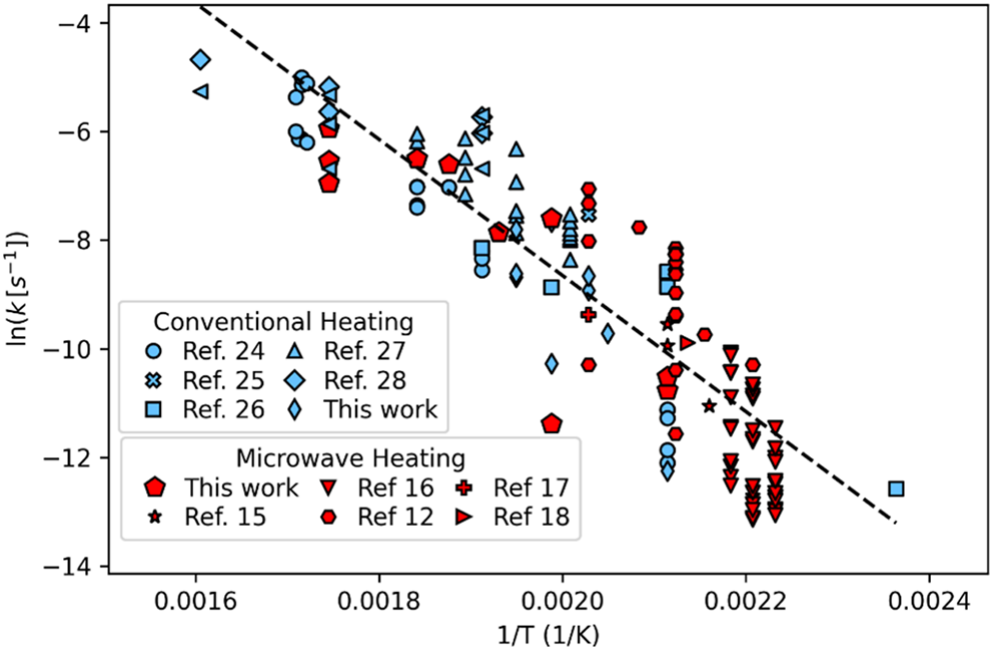
Arrhenius plot for PET conversion via hydrolysis with no added catalyst driven by conventional heating (blue) or microwave irradiation (red). Water/PET w/w loadings are between 8/1 and 10/1.^25,27,28^

The two data sets seem to show the same effect of temperature on the pseudo-first order rate constants. Fitting the entire data set to the Arrhenius equation provides an activation energy of 104 ± 5 kJ·mol^–1^ and ln­(*A* (s^–1^)) = 16.4 ± 1.1. These values are similar to those obtained by our previous work^24^ (118 ± 5 kJ·mol^–1^ and 18.8 ± 1.3, respectively) for PET hydrolysis driven by conventional heating. Fitting only the data set in Figure 3 from microwave heating results in Arrhenius parameters of *E*_a_ = 115 ± 10 kJ/mol and ln­(*A* (s^–1^)) = 19.0 ± 2.6, which are statistically indistinguishable from the values above published for PET hydrolysis with conventional heating.

Comparing the pseudo-first-order rate constants in Figure 3 provides one test of whether microwave irradiation affected the reaction rates observed for PET hydrolysis in neutral water with no added catalyst. This test was limited to using data solely from experiments done isothermally and using only the PET conversions. A more rigorous test would use a metric that included both PET conversion and TPA formation and accounted for any reaction that occurred during the nonisothermal heating and cooling of the reactor. These regions are especially important for “fast” hydrolysis reactions,^26^ which are completed within tens of seconds of batch holding time. Accordingly, the next section explores use of a metric that can correlate both conversion and TPA yield and easily handle results from both isothermal and nonisothermal reaction conditions.

#### 3.2.2 PET Hydrolysis—Comparing Product Yields at Identical Reaction Severities

The severity index is an empirical metric that originated in the field of biomass conversion.^30^ It is sometimes referred to as the Reaction Ordinate or Severity Factor. It combines the effects of reaction time and temperature into a single metric. It is especially convenient when examining together data from both isothermal and nonisothermal reactions. We have used the severity index (SI) in eq 2 in prior work^29^ on PET hydrolysis in neutral water with no added catalyst.

2 $$\mathrm{SI}=\int_{0}^{t}k\left(T_{\mathrm{ref}}\right)e^{-E_{a}/R\left(\left(1/T\right)-\left(1/T_{\mathrm{ref}}\right)\right)}dt$$

*k*(*T*_ref_) is the pseudo-first-order rate constant (2.36 × 10^–1^ s^–1^) at the reference temperature, *T*_ref_ = 700 K. *E*_a_ is an activation energy (taken as 1.18 × 10^5^ J/mol), *T* is temperature in K, *R* is the gas constant in J­(mol^–1^ K^–1^), and *t* is the batch holding time in seconds.

We use this severity index to compare TPA and PET yields from PET hydrolysis in neutral water with no added catalyst using microwave irradiation and conventional heating. This comparison includes results from both isothermal and nonisothermal conditions. Figure 4 displays the product yields displayed in Figure 2, Tables 1, and S1.

**Figure 4:**
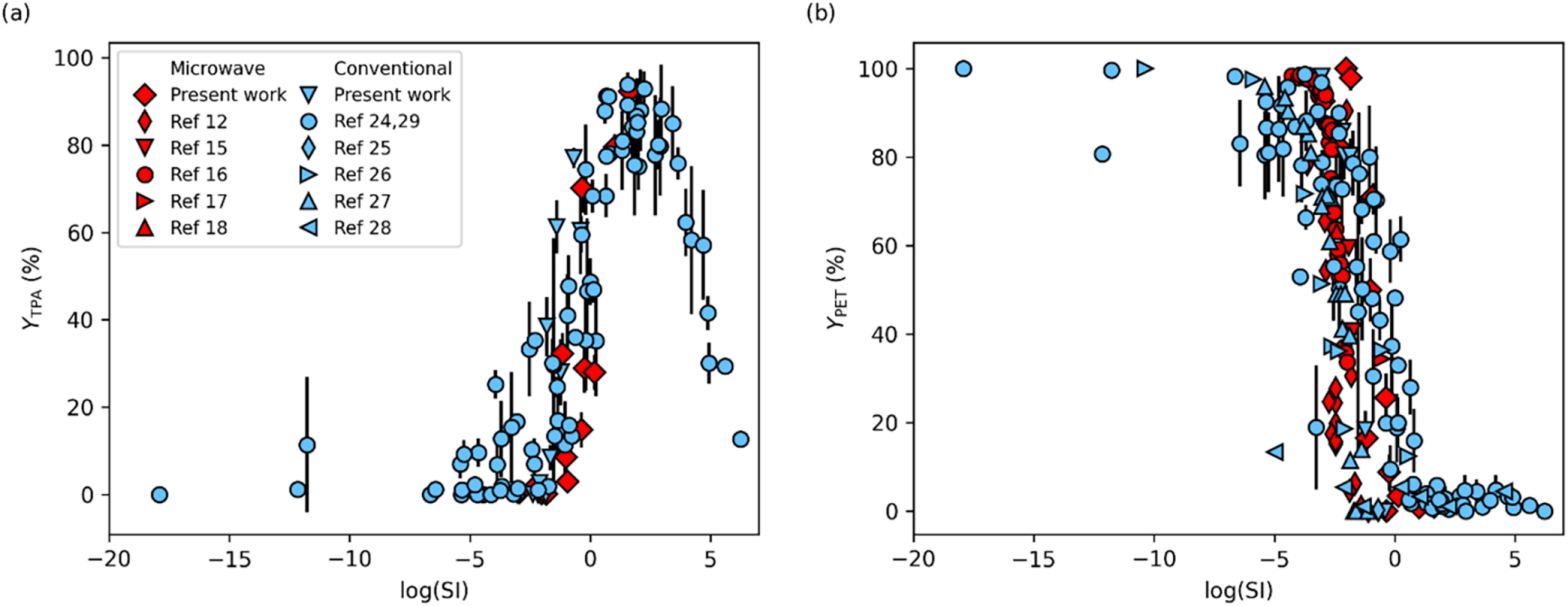
(a) TPA yield and (b) PET yield from PET hydrolysis with no added catalyst driven by conventional heating (blue) or microwave irradiation (red). Water/PET w/w loadings are between 8/1 and 10/1.

The TPA yields from the present experiments with microwave heating all fall within the yields observed previously for conventional heating at the same severity index (see Figure 4a). Likewise, Figure 4b shows the yields of PET remaining from microwave heating all fall within the yields observed previously for conventional heating at the same reaction severity. These product yields for PET hydrolysis appear independent of the heating method (microwave or conventional). The data show PET neutral hydrolysis with no added catalyst is not measurably influenced by nonthermal effects of microwaves (e.g., molecular reorientation and vibrational activation of polar groups).^31^

There appears to be no consistent difference between TPA yields and PET conversions from PET hydrolysis in water alone whether driven by conventional heating or microwave heating under the tested conditions. This outcome is reasonable, as PET absorbs microwaves poorly.^32^ Additionally, this finding is consistent with related work on hydrothermal liquefaction of biomass: Yang et al.^33^ examined liquefaction of spent coffee grounds in hot, compressed liquid water with no added catalyst using microwave irradiation and conventional heating. They found the yields of biocrude, biochar, and aqueous phase products were independent of the heating method. They also found the same was true for the biocrude composition and heating value, the biochar surface area and pore size, and the aqueous-phase pH and mineral content.

Our findings stand in contrast to Ikenaga et al. reporting measurable differences in TPA yields from PET hydrolysis done with conventional heating and with microwave heating at the same nominal conditions. To gather more information, we replicated the experiments reported by Ikenaga et al. using the Penn State microwave reactor. We used identical reactor loadings of water and PET (3.1/1 and 6.7/1 w/w water/PET). Ikenaga reported a TPA yield of 34.7% with microwave heating at 231 °C for 15 min. Our experiments led to TPA yields of 0.13 ± 0.10% and 0.35 ± 0.16% at the two PET/water ratios. The PET recoveries were 101.6 ± 1.1% and 95.3 ± 1.9%, respectively. These TPA yields are much lower than the 34.7% yield reported by Ikenaga et al. for nominally identical reaction conditions. Our new results are consistent with other published results (e.g., Table 1), whereas the yield reported by Ikenaga is much higher. We did not observe the large difference in TPA yield Ikenaga et al. reported for microwave vs conventional heating of PET hydrolysis.

### 3.3 PET Acetolysis

Just as water molecules can attack the ester linkages in PET, acetic acid molecules can do the same. Depolymerization produces TPA along with ethylene glycol diacetate. This approach to solvolytic depolymerization is only very recently receiving attention.^3–6^ Table 2 provides the first results from experiments using microwave irradiation for PET acetolysis (neat acetic acid) and for PET hydrolysis/acetolysis in mixtures of water and acetic acid. Acetic acid is a microwave absorber, like water, with a comparable loss tangent (0.17 at 25 °C).^34^

**Table 2:** Yields from PET Solvolysis with Conventional or Microwave Heating at 200 °C for 1 h

	*Y*_TPA_ (%)			*Y*_PET_ (%)		
acetic acid vol %	microwave heating	conventional heating	*p*-value	microwave heating	conventional heating	*p*-value
100	0.17 ± 0.18	<0.02	0.482^a^	89.5 ± 1.5	78.6 ± 2.3	0.002
44	6.25.5	1.94 ± 0.15	0.247^a^	66.8 ± 15.0	51.3 ± 17.0	0.289^a^
33	0.97 ± 0.23	0.04 ± 0.05	0.002	79.5 ± 6.4	77.5 ± 9.8	0.889^a^
20	<0.02	<0.02	1.000^a^	68.9 ± 5.5	61.9 ± 2.8	0.106^a^

a No statistically significant difference in yields (*p*-value >0.05).

PET depolymerization in acetic acid and water-acetic acid mixtures generally led to statistically insignificant differences in yields of TPA or undissolved solids. There are two instances where the differences were statistically significant, but the TPA yields were less than 1%. Thus, even though the differences in yield were significant in a statistical sense, the yields are too small for the differences to be significant in a practical sense. Since neutral hydrolysis of PET was unaffected by the heating method, we would anticipate the same outcome for acetolysis, as observed. Additional experimental work is required at higher temperatures and/or longer times to determine with this expectation is borne out at higher conversions.

### 3.4 Physical Appearance of Undissolved Solids

For a final comparison of microwave irradiation and conventional heating for PET hydrolysis, we offer Figure 5, which displays images of the undissolved solids from PET solvolysis experiments with conventional and microwave heating.

**Figure 5:**
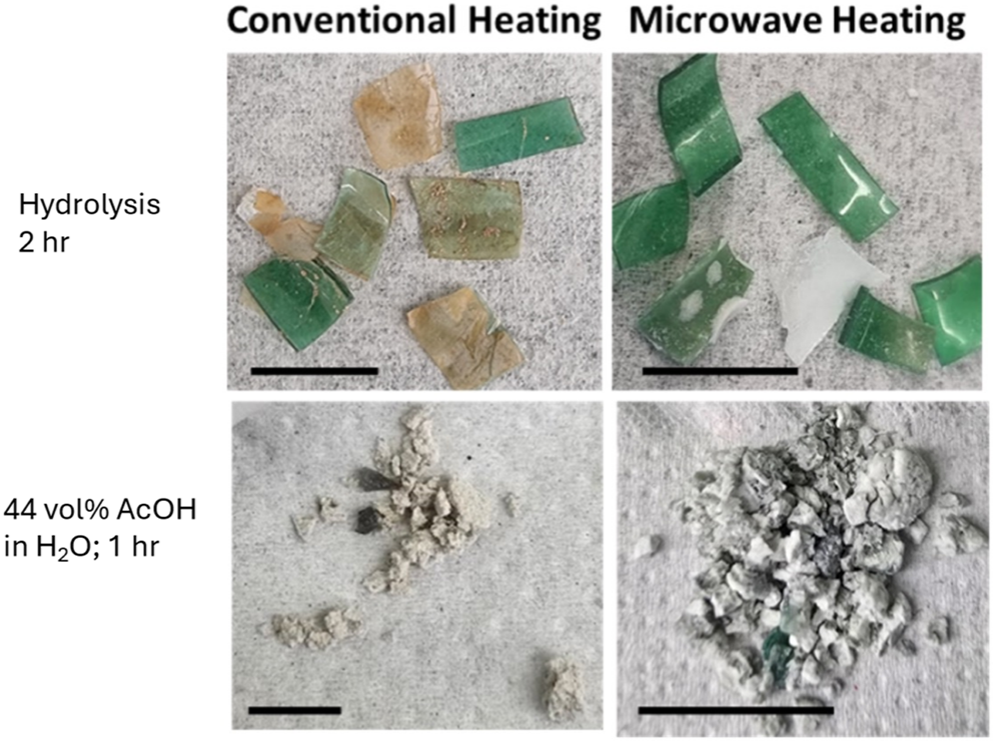
Images of undissolved solids from PET solvolysis experiments at 200 °C with microwave irradiation or conventional heating. Scale bars are equivalent to 5 mm.

For both solvolysis approaches, the undissolved solids produced from the two heating modes appear similar. The appearance is markedly different, however, for hydrolysis and for solvolysis in the mixed AcOH–H_2_O solvent. Though the reaction time was shorter for the acetic acid-containing solvent, the plastic bottle chips appear to have undergone significantly more degradation. This observation is consistent with the yield of unreacted PET being lower (around 60%) for the run with this solvent (Table 2) than for the hydrolysis run (93%). Since the yields of TPA were just a few percent in Table 2, the PET seems to have reacted to form oligomers that were soluble in DMSO but not detected in the HPLC analysis.

## 4 Summary and Conclusion

The yields of TPA and incompletely depolymerized PET from hydrolysis of PET in neutral water with no added catalyst were not affected in any statistically or practically significant manner by the energy input method (microwave radiation vs conventional conduction) at the conditions investigated (200–300 °C, 5–120 min, 8/1 to 10/1 w/w water/PET loading). These conditions spanned low conversion, intermediate conversion, and complete conversion. Likewise, the differences in product yields between microwave irradiation and conventional heating were negligible when using acetic acid, either pure or in an aqueous mixture for depolymerization. This indifference of the yields to the energy input mode is entirely reasonable. PET does not absorb microwave radiation, so the solvent would be the only microwave absorber in the system. For the depolymerization reaction, it is the fluid temperature that matters, not the modality of the energy input that provides the elevated temperature needed.

Alternate explanations exist for the two literature reports asserting nonthermal influences for microwave irradiation for PET hydrolysis with no added catalyst. The conclusion of nonthermal effects made by Zhang et al.^13^ could perhaps be an artifact due to the use of pseudo-first-order kinetics to model a system that is not truly first-order. We were not able to reproduce the different outcomes of the head-to-head comparison reported by Ikenaga et al.^11^ We observed the same very low TPA yield from microwave heating as expected from conventional heating. The reason for this discrepancy is not clear, but omitted details about the temperature–time history and experimental uncertainties in the Ikenaga et al. study might provide clues.

The conclusions drawn here are limited to PET hydrolysis and acetolysis under the conditions studied and with no added catalyst. Catalyzed solvolysis of PET is also of great interest. Careful and well-designed studies for catalyzed PET hydrolysis and acetolysis are needed to assess whether microwave irradiation can be used to advantage in these systems. Such work is underway in our laboratories.

## Supplementary Material



## Notes

Table with yields of TPA and PET conversion from many literature studies (PDF)
