# Supplementary material for: Driving Hydrolysis and Acetolysis of Poly(ethylene terephthalate) (PET) by Microwave and Thermal Energy Inputs: A Comparative Study
Source: ACS Omega. 2026 Feb 23;11(9):15062–70. doi: 10.1021/acsomega.5c12098 (PMC12980214; doi:10.1021/acsomega.5c12098)
Supplement: Supplementary file 1 [file ao5c12098_si_001.pdf]

SUPPORTING INFORMATION

Driving hydrolysis and acetolysis of polyethylene terephthalate (PET) by microwave and thermal energy inputs: A comparative study

Patrícia Pereira[a], Ashley C. Daniszewski[b], Matthew Staack[b], Emir Salmanzadeh[a], Hilal Ezgi Toraman[a,c, d*], Jianli Hu [b*], Christian W. Pester[a,e*], and Phillip E. Savage[a,d*]

[a] *Waltemeyer Department of Chemical Engineering, The Pennsylvania State University, University Park, PA 16802, United States.*

[b] *Department of Chemical and Biomedical Engineering, West Virginia University, Morgantown, WV 26505, United States*

[c] *Department of Energy and Mineral Engineering, The Pennsylvania State University, University Park, PA 16802, United States.*

[d] *Institute of Energy and the Environment, The Pennsylvania State University, University Park, PA 16801, United States*

[e] *Department of Materials Science and Engineering, University of Delaware, Newark, DE 19716, United States*

** Corresponding authors: H. Ezgi Toraman (hzt5148@psu.edu), John Hu (john.hu@mail.wvu.edu), Phillip Savage (psavage@psu.edu), Christian W. Pester (pester@udel.edu)*

Table S1. Yields of TPA and Unconverted PET from Hydrolysis of PET with no Added Catalyst and water/PET w/w loading from 8/1 to 10/1

Reaction Conditions	Energy Input	Y_{TPA} (%)	Y_{PET} (%)	ln(k [sec^{-1}])	Ref
200 °C, 210 min	Microwave	–	40.7	-9.5	15
200 °C, 180 min	Microwave	–	59.6	-9.9	15
180 °C, 180 min	Microwave	–	96.8	-12.7	15
190 °C, 180 min	Microwave	–	84.2	-11.0	15
175 °C, 90 min	Microwave	–	98.4	-12.7	16
175 °C, 120 min	Microwave	–	97.9	-12.7	16
175 °C, 150 min	Microwave	–	97.9	-13.0	16
175 °C, 180 min	Microwave	–	97.6	-13.0	16
175 °C, 210 min	Microwave	–	97.4	-13.1	16
175 °C, 240 min	Microwave	–	96.1	-12.8	16
175 °C, 270 min	Microwave	–	94.1	-12.5	16
175 °C, 300 min	Microwave	–	94.4	-12.7	16
175 °C, 330 min	Microwave	–	92.4	-12.4	16
175 °C, 360 min	Microwave	–	87.6	-12.0	16
175 °C, 390 min	Microwave	–	87.5	-12.1	16
175 °C, 420 min	Microwave	–	83.3	-11.8	16
175 °C, 450 min	Microwave	–	75.2	-11.5	16
180 °C, 90 min	Microwave	–	98.6	-12.9	16
180 °C, 120 min	Microwave	–	98.6	-13.1	16
180 °C, 150 min	Microwave	–	98.2	-13.1	16
180 °C, 180 min	Microwave	–	97.5	-13.0	16
180 °C, 210 min	Microwave	–	96.0	-12.6	16
180 °C, 240 min	Microwave	–	94.9	-12.5	16
180 °C, 270 min	Microwave	–	87.5	-11.7	16
180 °C, 300 min	Microwave	–	85.7	-11.7	16
180 °C, 330 min	Microwave	–	81.8	-11.5	16
180 °C, 360 min	Microwave	–	67.5	-10.9	16
180 °C, 390 min	Microwave	–	63.8	-10.9	16
180 °C, 420 min	Microwave	–	58.0	-10.7	16
180 °C, 450 min	Microwave	–	52.9	-10.7	16
185 °C, 90 min	Microwave	–	97.7	-12.4	16
185 °C, 120 min	Microwave	–	97.4	-12.5	16
185 °C, 150 min	Microwave	–	95.4	-12.2	16
185 °C, 180 min	Microwave	–	94.0	-12.1	16
185 °C, 210 min	Microwave	–	87.3	-11.4	16
185 °C, 240 min	Microwave	–	86.1	-11.5	16

185 °C, 270 min	Microwave	–	73.9	-10.9	16
185 °C, 300 min	Microwave	–	59.2	-10.4	16
185 °C, 330 min	Microwave	–	55.9	-10.4	16
185 °C, 360 min	Microwave	–	53.1	-10.4	16
185 °C, 390 min	Microwave	–	37.1	-10.1	16
185 °C, 420 min	Microwave	–	35.8	-10.1	16
185 °C, 450 min	Microwave	–	33.7	-10.1	16
220 °C, 50 min	Microwave	–	90.4	-10.3	12
220 °C, 60 min	Microwave	–	30.6	-8.0	12
220 °C, 70 min	Microwave	–	6.2	-7.3	12
220 °C, 90 min	Microwave	–	1.0	-7.1	12
220 °C, 120 min	Microwave	–	0.0	–	12
T_{sat} = 198 °C, 120 min	Microwave	–	24.5	-8.5	12
T_{sat} = 198 °C, 120 min	Microwave	–	19.8	-8.4	12
T_{sat} = 198 °C, 120 min	Microwave	–	15.6	-8.3	12
T_{sat} = 198 °C, 120 min	Microwave	–	27.7	-8.6	12
T_{sat} = 198 °C, 120 min	Microwave	–	54.8	-9.4	12
T_{sat} = 198 °C, 50 min	Microwave	–	97.2	-11.6	12
T_{sat} = 198 °C, 60 min	Microwave	–	89.5	-10.4	12
T_{sat} = 198 °C, 70 min	Microwave	–	69.8	-9.4	12
T_{sat} = 198 °C, 80 min	Microwave	–	54.3	-9.0	12
T_{sat} = 198 °C, 90 min	Microwave	–	24.7	-8.3	12
T_{sat} = 198 °C, 100 min	Microwave	–	17.5	-8.1	12
T_{sat} = 180 °C, 120 min	Microwave	–	78.4	-10.3	12
T_{sat} = 191 °C, 120 min	Microwave	–	65.5	-9.7	12
T_{sat} = 207 °C, 120 min	Microwave	–	4.6	-7.8	12
T_{sat} = 198 °C, 120 min	Microwave	–	15.1	-8.2	12
T_{sat} = 198 °C, 120 min	Microwave	–	15.7	-8.3	12
T_{sat} = 198 °C, 120 min	Microwave	–	15.8	-8.3	12
220 °C, 210 min	Microwave	–	34.4	-9.4	17
195 °C, 150 min	Microwave	–	63.2	-9.9	18
220 °C, 180 min	Thermal	–	0.3	-7.5	25
100 °C, 120 min	Thermal	–	100	–	26
150 °C, 120 min	Thermal	–	97.6	-12.6	26
200 °C, 30 min	Thermal	–	71.7	-8.6	26
200 °C, 60 min	Thermal	–	51.4	-8.6	26
200 °C, 90 min	Thermal	–	37.2	-8.6	26
200 °C, 120 min	Thermal	–	36.2	-8.9	26
200 °C, 150 min	Thermal	–	18.6	-8.6	26
230 °C, 120 min	Thermal	–	36.4	-8.9	26

250 °C, 120 min	Thermal	–	12.4	-8.1	26
225 °C, 3 min	Thermal	–	95.9	-8.4	27
225 °C, 5 min	Thermal	–	90.4	-8.0	27
225 °C, 10 min	Thermal	–	81.0	-8.0	27
225 °C, 15 min	Thermal	–	71.0	-7.9	27
225 °C, 20 min	Thermal	–	61.0	-7.8	27
225 °C, 25 min	Thermal	–	49.0	-7.7	27
225 °C, 30 min	Thermal	–	49.0	-7.5	27
225 °C, 50 min	Thermal	–	0.0	–	27
240 °C, 3 min	Thermal	–	93.3	-7.9	27
240 °C, 5 min	Thermal	–	85.3	-7.5	27
240 °C, 10 min	Thermal	–	70.8	-7.5	27
240 °C, 15 min	Thermal	–	41.2	-6.9	27
240 °C, 20 min	Thermal	–	11.5	-6.3	27
240 °C, 25 min	Thermal	–	0.0	–	27
255 °C, 3 min	Thermal	–	86.9	-7.2	27
255 °C, 5 min	Thermal	–	71.4	-6.8	27
255 °C, 10 min	Thermal	–	39.7	-6.5	27
255 °C, 15 min	Thermal	–	14.0	-6.1	27
255 °C, 20 min	Thermal	–	0.0	-	27
270 °C, 3 min	Thermal	–	68.9	-6.2	27
270 °C, 5 min	Thermal	–	49.1	-6.0	27
270 °C, 10 min	Thermal	–	0.0	–	27
250 °C, 10 min	Thermal	–	13.4	–	28
250 °C, 20 min	Thermal	–	5.4	-5.7	28
250 °C, 60 min	Thermal	–	1.1	-6.0	28
300 °C, 10 min	Thermal	–	5.4	-5.2	28
300 °C, 20 min	Thermal	–	3.3	-5.6	28
300 °C, 60 min	Thermal	–	1.1	-4.7	28
350 °C, 10 min	Thermal	–	4.5	–	28
200 °C, 120 min	Thermal	–	90.00	-11.1	24
250 °C, 30 min	Thermal	–	60.91	-8.2	24
270 °C, 30 min	Thermal	–	19.88	-7.0	24
310 °C, 30 min	Thermal	86.7 ± 3.8	2.2 ± 0.8	-6.1	24
310 °C, 10 min	Thermal	77.5 ± 1.4	2.9 ± 1.2	-5.1	24
200 °C, 120 min	Thermal	6.9 ± 6.0	85.4 ± 3.3	-10.7	24
250 °C, 30 min	Thermal	15.9 ± 0.7	70.6 ± 3.6	-8.6	24
270 °C, 30 min	Thermal	40.4 ± 3.8	20.0 ± 5.6	-7.0	24
270 °C, 120 min	Thermal	89.2 ± 3.5	0.7 ± 0.2	-7.4	24
310 °C, 10 min	Thermal	91.3 ± 1.7	1.8 ± 0.9	-5.0	24

310 °C, 30 min	Thermal	83.1 ± 6.3	2.2 ± 1.7	–	24
311 °C, 30 min	Thermal	85.2 ± 1.9	1.6 ± 1.7	-6.1	24
200 °C, 120 min	Thermal	2.1 ± 0.9	89.9 ± 6.0	-11.1	24
200 °C, 120 min	Thermal	2.8 ± 0.1	91.3 ± 0.4	-11.3	24
200 °C, 30 min	Thermal	0.9 ± 0.8	98.9 ± 0.3	-12.1	24
250 °C, 30 min	Thermal	–	65.0	-8.3	24
260 °C, 30 min	Thermal	59.6 ± 4.2	20.0 ± 0.2	-7.0	24
270 °C, 30 min	Thermal	–	33.0	-7.4	24
308 °C, 10 min	Thermal	87.8 ± 2.8	2.6 ± 0.5	-6.2	24
308 °C, 30 min	Thermal	75.5 ± 11.6	2.60 ± 1.7	-5.1	24
300 °C, 15 sec	*Thermal	0 ± 0	100.0 ± 0.0	–	29
300 °C, 60 sec	*Thermal	0 ± 0	98.3 ± 0.0	–	29
300 °C, 85 sec	*Thermal	0 ± 0	95.9 ± 0.0	–	29
300 °C, 115 sec	*Thermal	0 ± 0	96.9 ± 3.4	–	29
300 °C, 175 sec	*Thermal	2 ± 1	78.7 ± 7.4	–	29
350 °C, 55 sec	*Thermal	0 ± 0	92.5 ± 0.0	–	29
350 °C, 60 sec	*Thermal	0 ± 0	91.7 ± 0.0	–	29
350 °C, 65 sec	*Thermal	0 ± 0	86.9 ± 0.0	–	29
350 °C, 75 sec	*Thermal	0 ± 0	90.3 ± 3.0	–	29
350 °C, 115 sec	*Thermal	11 ± 10	80.1 ± 11.6	–	29
350 °C, 175 sec	*Thermal	35 ± 13	61.5 ± 5.1	–	29
400 °C, 45 sec	*Thermal	1 ± 1	86.7 ± 8.7	–	29
400 °C, 55 sec	*Thermal	2 ± 1	88.2 ± 6.9	–	29
400 °C, 60 sec	*Thermal	1 ± 0	78.9 ± 2.7	–	29
400 °C, 65 sec	*Thermal	10 ± 1	73.6 ± 11.7	–	29
400 °C, 75 sec	*Thermal	13 ± 3	76.2 ± 13.8	–	29
400 °C, 85 sec	*Thermal	13 ± 1	70.3 ± 12.2	–	29
400 °C, 95 sec	*Thermal	35 ± 12	58.8 ± 7.2	–	29
400 °C, 115 sec	*Thermal	68 ± 5	28.0 ± 6.3	–	29
400 °C, 175 sec	*Thermal	87 ± 9	1.9 ± 1.0	–	29
450 °C, 45 sec	*Thermal	7 ± 5	78.1 ± 8.4	–	29
450 °C, 55 sec	*Thermal	1 ± 0	72.8 ± 9.6	–	29
450 °C, 60 sec	*Thermal	30 ± 29	45.1 ± 32.8	–	29
450 °C, 65 sec	*Thermal	41 ± 9	48.1 ± 1.9	–	29
450 °C, 75 sec	*Thermal	49 ± 5	48.2 ± 1.0	–	29
450 °C, 105 sec	*Thermal	84 ± 8	5.8 ± 1.5	–	29
450 °C, 135 sec	*Thermal	78 ± 14	3.4 ± 1.7	–	29
450 °C, 175 sec	*Thermal	85 ± 8	4.4 ± 2.7	–	29
480 °C, 35 sec	*Thermal	7 ± 3	80.4 ± 10.0	–	29
480 °C, 45 sec	*Thermal	17 ± 1	73.9 ± 4.0	–	29

480 °C, 55 sec	*Thermal	25 ± 2	68.2 ± 3.2	–	29
480 °C, 65 sec	*Thermal	47 ± 17	37.3 ± 11.8	–	29
480 °C, 75 sec	*Thermal	78 ± 2	15.9 ± 7.1	–	29
480 °C, 95 sec	*Thermal	88 ± 9	2.9 ± 2.7	–	29
480 °C, 115 sec	*Thermal	80 ± 11	4.7 ± 3.5	–	29
480 °C, 175 sec	*Thermal	58 ± 17	4.9 ± 3.3	–	29
500 °C, 35 sec	*Thermal	1 ± 0	83.1 ± 9.8	–	29
500 °C, 45 sec	*Thermal	33 ± 11	55.3 ± 12.3	–	29
500 °C, 60 sec	*Thermal	74 ± 10	9.5 ± 5.4	–	29
500 °C, 75 sec	*Thermal	79 ± 9	4.0 ± 1.2	–	29
500 °C, 175 sec	*Thermal	57 ± 13	3.3 ± 2.6	–	29
510 °C, 35 sec	*Thermal	10 ± 3	81.9 ± 10.8	–	29
510 °C, 45 sec	*Thermal	35 ± 1	50.2 ± 10.5	–	29
510 °C, 55 sec	*Thermal	36 ± 2	43.2 ± 4.6	–	29
510 °C, 75 sec	*Thermal	94 ± 3	0.7 ± 0.6	–	29
510 °C, 95 sec	*Thermal	80 ± 6	1.4 ± 0.6	–	29
510 °C, 115 sec	*Thermal	76 ± 4	1.0 ± 0.3	–	29
510 °C, 175 sec	*Thermal	30 ± 5	0.8 ± 1.0	–	29
540 °C, 35 sec	*Thermal	25 ± 3	53.0 ± 1.9	–	29
540 °C, 45 sec	*Thermal	30 ± 6	55.2 ± 5.4	–	29
540 °C, 55 sec	*Thermal	68 ± 4	18.8 ± 7.8	–	29
540 °C, 65 sec	*Thermal	81 ± 3	1.7 ± 0.5	–	29
540 °C, 75 sec	*Thermal	93 ± 4	0.5 ± 0.5	–	29
540 °C, 85 sec	*Thermal	88 ± 10	0.0 ± 0.0	–	29
540 °C, 105 sec	*Thermal	62 ± 8	2.4 ± 1.0	–	29
540 °C, 135 sec	*Thermal	42 ± 4	3.2 ± 2.1	–	29
540 °C, 175 sec	*Thermal	29 ± 1	1.3 ± 0.1	–	29
550 °C, 15 sec	*Thermal	1 ± 0	80.8 ± 0.5	–	29
550 °C, 30 sec	*Thermal	9 ± 3	81.1 ± 9.0	–	29
550 °C, 35 sec	*Thermal	13 ± 9	66.4 ± 2.8	–	29
550 °C, 45 sec	*Thermal	17 ± 9	50.2 ± 11.7	–	29
570 °C, 15 sec	*Thermal	11 ± 15	99.6 ± 0.9	–	29
570 °C, 30 sec	*Thermal	2 ± 1	86.3 ± 12.0	–	29
570 °C, 35 sec	*Thermal	15 ± 13	19.0 ± 14.0	–	29
570 °C, 45 sec	*Thermal	48 ± 7	30.5 ± 10.5	–	29
570 °C, 175 sec	*Thermal	13 ± 1	0.0 ± 0.0	–	29

* Temperature given is heat source set point temperature. The reactor rapidly heats from ambient toward this temperature but does not reach it before the reaction is terminated.
